# HTKS-Kids: A tablet-based self-regulation measure to equitably assess young children's school readiness

**DOI:** 10.3389/fpsyg.2023.1202239

**Published:** 2024-01-11

**Authors:** Claire E. Cameron, Megan M. McClelland, Tammy Kwan, Krystal Starke, Tanya Lewis-Jones

**Affiliations:** ^1^Department of Learning and Instruction, University at Buffalo, Buffalo, NY, United States; ^2^School of Human Development and Family Sciences, Oregon State University, Corvallis, OR, United States; ^3^Cognitive ToyBox, Inc., New York, NY, United States

**Keywords:** early childhood, equity, executive function, school readiness, self-regulation, technology-based assessment

## Abstract

**Background:**

Technology advances make it increasingly possible to adapt direct behavioral assessments for classroom use. This study examined children's scores on HTKS-Kids, a new, largely child-led version of the established individual research assessment of self-regulation, Head-Toes-Knees-Shoulders-Revised task (HTKS-R). For the HTKS-Kids tablet-based assessment, which was facilitated by children's preschool teachers, we examined (1) preliminary reliability and validity; (2) variation in scores predicted by child age and background characteristics; and (3) indication that HTKS-Kids provides different information from teacher ratings of children.

**Method:**

Participants included *n* = 79 4-year-old children from two urban areas in upstate New York, USA. Average parent education was 12.5 years, ranging 3–20. A researcher administered the HTKS-R to individual children, and teachers (eight white, two Latino) were trained to use the HTKS-Kids tablet-based assessment and asked to play once with each study child. Teachers also rated each child on 10 Child Behavior Rating Scale (CBRS) items about classroom self-regulation.

**Results:**

We found evidence that (1) the HTKS-Kids captures variation in children's self-regulation and correlates positively with established measures, (2) parent education was the best predictor of HTKS-Kids scores, and (3) teachers rated Black children significantly worse and white children better on the CBRS, with the magnitude of group differences similar to the contribution of parent education. In contrast, Black and white children showed no score differences on HTKS-Kids.

**Implications:**

The HTKS-Kids is a promising new tablet-based assessment of self-regulation that could replace or supplement traditional teacher ratings, which are often subject to implicit bias.

## Introduction

Developmentally appropriate assessment is part of supporting young children's successful transition to school (Hirsh-Pasek et al., 2005). Assessment includes any tool or method that helps teachers or educational systems document children's knowledge and skills (Neuman and Devercelli, 2013; Smith et al., 2015). In early childhood, assessment has many purposes, such as to inform learning activities, to identify or screen individual children for intervention in an area such as speech or motor skills, or to improve educational programs and curricula through evaluation (National Association for the Education of Young Children and the National Association of Early Childhood Specialists in State Departments of Education, 2003; Gokiert et al., 2013, p. 1). Experts consider holistic assessment in multiple skill domains to be developmentally appropriate for young children and can support their learning by more precisely identifying their needs and strengths (Hirsh-Pasek et al., 2005; National Research Council, 2008).

Holistic child assessment includes academic as well as non-academic skills and is recognized as critical, given the intertwined nature of multiple developmental domains in the early childhood period (McClelland and Cameron, 2018). Experts recommend naturalistic and observation-based assessment approaches but these can pose significant burdens for teachers (Cameron et al., 2023). Ideal assessment practices are not always implemented however, and teachers, who may be short of time, may inadvertently introduce bias or error in their documentation of children's skills (Waterman et al., 2012). Structured direct assessment is one alternative to naturalistic observations where an adult presents an individual child with tasks or questions. Direct assessment is more standardized, which can increase the reliability and validity of assessment (National Research Council, 2008); and allows for the development of reports and recommendations to compare data across children, classrooms and programs (Waterman et al., 2012). In particular, technology-assisted direct assessment has the potential to increase the frequency of assessment, feedback to students and teachers, objectivity and consistency, and administrative efficiency (Bull and McKenna, 2003). In this study, we report preliminary psychometric properties from a tablet-based adaptation of an internationally-used direct research assessment of self-regulation, the Head-Toes-Knees-Shoulders-Revised or HTKS-R (Gonzales et al., 2021; McClelland et al., 2021). The new measure, HTKS-Kids, is a tablet assessment with highly similar regulatory demands to the HTKS-R research instrument. HTKS-Kids is teacher-facilitated but largely child-led, and is designed to be integrated into the regular preschool day.

### Assessment of self-regulation in early childhood

The preschool years are a critical time for children's development, particularly as they learn to self-regulate, or to effectively manage their nervous system, emotions, cognition, and behaviors across contexts (Bailey and Jones, 2019; Blair and Ku, 2022). Self-regulation has its roots in infant attention and reactivity (Rothbart et al., 2006) and changes throughout development (McClelland et al., 2015b). Self-regulatory skills among typically developing children improve dramatically in early childhood as the prefrontal cortex matures to support executive function (McClelland and Cameron, 2012; Bailey and Jones, 2019). Executive function (EF) is an umbrella term that describes the specific cognitive processes associated with developing, planning, and executing goals (Miyake et al., 2000).

As students progress through school, their self-regulatory capacities grow with advances in EF, enabling them to plan and carry out increasingly complex task sequences (Blair and Raver, 2015). In early childhood, where this study focuses, strong EF allows children to intake, process, filter and organize information; discard extraneous information; and make adaptive choices (Diamond, 2016). By the end of early childhood, EF processes that can be measured distinctly include working memory, task-switching, and inhibitory control; which work together to contribute to overall self-regulation of behavior and responses in a given environment such as a classroom (Blair and Ku, 2022). Both constructs predict future academic and personal success, with EF more closely associated with cognitive processes that are consciously applied, and self-regulation encompassing adaptations to environments that may or may not allow for the practice and exercise of EF (Zelazo, 2020; Blair and Ku, 2022). Children's performance on measures of both self-regulation and EF are strong predictors of their school readiness (Blair, 2002; McClelland et al., 2014), as well as math and verbal abilities (Blair and Razza, 2007). EF is linked to abstract thinking and problem-solving, otherwise known as fluid intelligence (Blair, 2006). More adaptive self-regulation at age 4 is directly linked to greater academic achievement starting at 7 years old through adulthood (McClelland et al., 2013). Children with strong self-regulation skills are also less likely to engage in criminal or problematic behavior as a young person or adult (Moffitt et al., 2011). In sum, EF is a set of cognitive skills that facilitates learning as well as adaptive self-regulation, which is a broader term that encompasses cognitive skills but also refers to children's functioning in social contexts (Bailey and Jones, 2019; Blair and Ku, 2022).

Many research measures of self-regulation as well as EF exist for children under 5 years (Lipsey et al., 2017; McClelland et al., 2022). Strong measures need to be developmentally appropriate, ecologically relevant, and demonstrate strong psychometric properties. Historically, EF had been evaluated in children by using adult assessments extended downward for use in children (Gnys and Willis, 1991; Delis et al., 2001). Adult-derived tests, however, did not sufficiently evaluate children's skills and the content was not always relevant to children (Anderson, 1998). Furthermore, traditional cognitive measures of EF were developed in highly controlled laboratory-based settings, which often do not reflect the more dynamic and diffuse self-regulatory demands on children in less formal settings (Salthouse et al., 2003). On the other hand, in schools, self-regulation is typically measured with observer-report checklists or surveys (Zelazo et al., 2016). As the literature has expanded emphasizing the importance of both EF and self-regulation for young children, it is increasingly recognized that measures must be appropriate for use in educational or other naturalistic settings (Franzen and Wilhelm, 1996; McClelland and Cameron, 2012). While many new instruments to measure EF and self-regulation as part of social-emotional learning (SEL) have been developed for children at the formal school transition (Carlson, 2005; Denham et al., 2010), few direct assessments are available for non-researchers.

Overall, improvements in assessment strengthen reliability and validity of an instrument for a specific population or age group. Reliability indicates whether an assessment is consistent, that is, whether different items measure the same underlying construct; while validity refers to whether the assessment measures the knowledge, skills, or capacities that it is designed to measure (Arizmendi et al., 1981; Hartmann and Pelzel, 2015). There are different ways to demonstrate a measure's validity, including correlations with established instruments that measure the same construct; investigation of demographic or other characteristics known to explain variance in the construct; and correlations with other constructs that are related to, but not the same as, the construct of focus (Cameron Ponitz et al., 2008; Gonzales et al., 2021).

### Importance of direct assessment options for equity in early childhood

While developmentally-appropriate measures have improved accuracy in capturing skill levels, measuring individual differences remains a challenge, especially for children whose regulatory skill development is nascent (Willoughby et al., 2012; Gonzales et al., 2021). Furthermore, recent efforts have focused on measuring these skills in a way that is fair and equitable in a society where systemic oppression limits opportunities for Black, brown, and poor people (Miller-Cotto et al., 2022). For example, Miller-Cotto et al. note that current conceptualizations and measurement of EF and self-regulation are rooted in decades of research on primarily white children from privileged backgrounds. They urge the “repositioning of executive functions as skills developed through task–environment exchanges” (p. 6). Part of this effort means recognizing that how EF develops and even how it is measured is itself a cultural enterprise, which mostly white and privileged researchers have historically overseen.

With equity goals in mind, we draw from prior research showing that EF and self-regulation improve with age, especially in the years from 3 to 5 (Garon et al., 2008). The research base also indicates that children from impoverished communities, and those whose parents have obtained less education, demonstrate lower levels of self-regulation and EF compared with same-age children from more resourced backgrounds, whose parents tend to have higher levels of education (Ursache et al., 2016). These patterns have been linked to opportunities to develop and practice EF, which are more common in high-resource homes (Blair and Raver, 2015). Of note, cultural group membership and socio-economic status (SES) are closely intertwined given the history of power in the U. S. Historical context must be acknowledged when assessing children's regulatory capacities, and when developing new assessments (Miller-Cotto et al., 2022). Historically and today, people racialized as white retain power, privilege, and resources that lead to greater opportunity, on average, than people who are racialized as non-white. This reality necessitates intentionality in developing assessments that have the potential to uncover assets held by children who have been minoritized. Miller-Cotto et al. urge researchers to bring assessment out of historically white spaces, such as the laboratory, and to “celebrate children's ability to persist through real-world distractions and perform complex, planful actions in rapidly changing environments” (p. 9). In other words, researchers must strive to measure children's skills where they are relevant.

This study introduces a measure of self-regulation drawing on EF that can be used within the school context. We acknowledge that like laboratories, U. S. education settings tend to be white-dominated, where assessing self-regulation and EF is a culturally-embedded activity with serious implications for non-white and poor children (Miller-Cotto et al., 2022). Although differences in self-regulation scores by child characteristics may be expected because children have different experiences, as well as energy or attention levels, researchers caution that differences commonly arise from factors outside the child (Mashburn et al., 2006). Equitable early childhood assessment can minimize the extent to which non-child factors contribute to score differences.

Teacher ratings of children's behavior and self-regulatory skills provide a comprehensive view of a student and are used ubiquitously in schools to identify children for behavioral intervention, disciplinary action, and instructional needs. Teacher ratings can be accurate, especially when identifying students in need of academic intervention (Gresham et al., 1987). There is growing evidence, however, that teacher bias also exists and has a significant impact on long term academic outcomes for students (Reardon et al., 2017). Waterman et al. (2012) report that preschool teachers, as compared to extramural research assessors, appear prone to significant bias when rating children's skills.

Social reproduction theory helps to explain how schools replicate social inequalities, particularly racial inequality (Dixon and Rousseau, 2005). These inequalities are exacerbated by racial, ethnic, and cultural misunderstandings between teachers and students (Boykin, 1986; Delpit, 2006). In their study of 701 prekindergarten students across 11 states, Downer et al. (2016) found that Black students are more often the recipients of escalating disciplinary action by white teachers over time. Black students are more often recommended for special education services by white teachers than by Black teachers (Wiley et al., 2013). Implicit bias is the unconscious opinions or attitudes held against different social groups, and implicit bias influences student outcomes in schools (Glock and Kovacs, 2013). The impact of implicit bias can be significant, with teachers' beliefs about student performance resulting in self-fulfilling prophesies for students of color (Papageorge et al., 2016). Some evidence indicates that compared to white teachers, Black teachers hold higher expectations for Black students, and this contributes to more positive outcomes for all students (Gregory et al., 2011).

As another example, gender differences are common in teacher ratings, more so than in direct assessments. Differences usually favor girls (Matthews et al., 2009; Wanless et al., 2013), which some experts attribute to the greater alignment of girls' behavior in classrooms with teacher expectations (Entwisle et al., 2007). In addition to identifying as white or Caucasian, most early childhood teachers are also women. Teachers from multiple countries including the U. S. tend to report that girls have higher classroom self-regulation (Wanless et al., 2011b).

If group-based differences appear in teacher ratings but not direct assessments, that gap raises questions about differences in the assessment contexts and in the individuals responsible for the assessment. Given the pervasiveness of teacher bias in educational systems, multiple modes of assessment including ratings and direct measures can be employed to provide a more holistic, and potentially more equitable, evaluation of children.

### Technology as part of early childhood assessment

In this study we explored an alternative to teacher ratings by adapting a child-friendly research-based measure of self-regulation, through digital technology. Tablet use by children, particularly the use of tablets in all facets of education, has grown over the past few decades (Fletcher et al., 2014). Furthermore, touchscreens have eased technology use for young children (Christakis, 2014; Spawls and Wilson, 2017) and as a result preschool children use technology frequently (Vandewater et al., 2007; Rideout and Katz, 2016). Furthermore, recent studies show that instruction using tablets can be beneficial to enhancing early childhood learning alphabet awareness (De Jong and Bus, 2004; Xie et al., 2018; Griffith et al., 2020) and numeracy support (Outhwaite et al., 2017). Overall, experts recommend that digital technology can be effectively incorporated as part of instructional programming that is of high quality and age appropriate.

The adoption of digital technology in preschool classrooms can only occur with preparedness and engagement of educators. Before COVID-19, teachers were expected to use digital technology as at least a supplement to traditional classroom instruction (Collier et al., 2004; Hernández-Ramos, 2005). Large-scale studies highlight the increase in technology use in classrooms (Barron et al., 2003; Carson et al., 2014; Denham et al., 2020). Teachers are finding technology-based testing easy to administer and there is research to support the benefits of technology-based testing over traditional tests (Tymms, 2001; Martin, 2008). Young students typically enjoy using tablets in particular, and are able to negotiate the use of digital technology with relative ease (Jones and Liu, 1997). Technology-based assessment reduces the time and effort required to administer and score the assessment, as well as to train testing examiners (Denham et al., 2020).

### Rationale for the present study

This study introduces the HTKS-Kids tablet-based assessment of self-regulation, requiring EF. In early childhood education settings, observation-based assessment and behavioral rating scales remain the most common approach to assess school readiness, including EF and self-regulation (Schilder and Carolan, 2014; Isaacs et al., 2015). Direct measures of self-regulation have blossomed in research settings but are still not widely available for preschool programs. Research suggests that both teacher rating scales and direct assessments can predict children's outcomes measured longitudinally (Schmitt et al., 2014). Given that all assessment, including technology-based assessment, is culturally-embedded with potential for equity or bias, it is imperative to understand how a new teacher-facilitated, largely child-led technology-based assessment of self-regulation captures children's skills in a diverse sample. HTKS-Kids is child-friendly and the concept of touching the opposite (head vs. toes) is based on a game designed for naturalistic settings (McCabe et al., 2004), as opposed to laboratory tasks that prioritize standardized administration. Thus, HTKS-Kids may have advantages over other tablet-based EF measures with origins in the laboratory, which may have more rigid administration requirements (Carlson and Zelazo, 2014).

In this study, we compared a new tablet-based direct assessment of self-regulation with the original research task and teacher-rated classroom self-regulation, keeping implications for equity in mind. We examined preliminary psychometric properties, including measure variability, reliability, and validity; potential sources of difference in children's scores; and finally, evidence that the tablet-based measure provides different information from teacher ratings. We posed the following research questions:

1. Does HTKS-Kids show preliminary validity and reliability as a measure of self-regulation among low-income 4-year-old preschoolers, when compared with the established HTKS-R research measure of self-regulation?
2. How much variation in children's HTKS-Kids scores is due to key background and sociocultural characteristics, including their age, gender, parent education, first language, and ethnicity identified by parents (Black, Latino/a, or white)?
3. What is the association between children's HTKS-Kids scores and teacher ratings of their classroom self-regulation, and do these two measures provide different information, focusing on key child characteristics (gender and ethnicity)?

## Method

The present research questions were posed in the context of a short-term, cross-sectional study lasting from October 2021 to February 2022. We report data collected with children and families including teacher ratings of children (Teddlie and Tashakkori, 2009).

### Teacher sample

At study enrollment, teachers completed a demographic survey on Qualtrics. Two identified as male and nine as female; nine reported their primary/only ethnic group as white with two selecting Latino/a. All but one teacher reported their age range as 26–39 years, with one reporting 40–49 years. Seven teachers had 6–9 years of experience teaching preschool, and the other 4 had 10 or more years. They taught in three different programs in upstate New York, with one person being the only participant from their program.

### Child sample

Children (*n* = 79) were on average 4.4 years old on November 1st, ranging from 3.9 to 4.9 years. The sample was 54% female and 78% of families reported qualifying for the WIC subsidy. The average years of parent education was 12.5, or just over a high school degree, ranging from 3 to 20. Families were asked to identify the child's ethnicity and could endorse as many groups as they liked. Of 78 families reporting this variable, the sample included *n* = 34 or 39.5% of families who endorsed Black or multiethnic Black, *n* = 21 or 26.9% Latino (non-Black), *n* = 17 or 21.8% White only; with other groups representing 5% or less of the sample including American Indian, Asian, and Middle Eastern. English was reported as the child's first language for 70 or 81.4% of participants; other first languages included Spanish (*n* = 8), Arabic (*n* = 3), Burmese (*n* = 1), and Nepali (*n* = 1).

### Procedures

Participating teachers sent backpack mail and electronic flyers with the demographic questionnaire home to families, resulting in 86 children enrolled in the study. A total of seven children dropped from their preschool programs before the study was completed. Sample reported in this paper range from 71 to 79 children depending on how many study measures were available. The majority of data were collected in late November and December, with data collection complete by mid-February.

Either the PI or research assistant administered the research assessment, HTKS-R (Gonzales et al., 2021; McClelland et al., 2021) to individual children in a quiet hallway or office near the child's classroom. There were no experimenter differences in mean score obtained (*t*_72_ = 1.00, *p* = 0.3). Teachers facilitated the HTKS-Kids measure with all their study children. About half of teachers left the classroom to work with individual children, to facilitate their engagement, and the other half administered HTKS-Kids to individual children in the classroom, during center time. About half (46%) of the sample were given the HTKS-R first; the remaining 54% of children took the traditional research version of the HTKS-R after they had played the HTKS-Kids version on the tablet with their teachers. Finally, teachers rated each children's classroom self-regulation. Most teachers completed the CBRS on paper, though it was also available electronically.

### Measures

We collected several instruments one time on each child.

#### Child demographics

Children's primary caregivers completed a demographic survey for their child upon entry into the study. These were done on paper or electronically. We obtained demographic information for 85 children.

#### Traditional HTKS-R assessment

In the first part, called Opposites, children were told to say “head” if the examiner says “toes” and vice versa. Then children were asked to “touch your head” if told to touch their toes and vice versa. Children who did well on the “touch” commands advanced and were taught to touch knees when told to touch shoulders and vice versa, with one of four commands being given (head, toes, knees, shoulders). Finally, if they did well on that part, the rules switched and they were taught to touch their head when told to touch their knees, and touch their toes when told to touch their shoulders. HTKS-R items were scored 0 (incorrect), 1 (self-correct), or 2 (correct); with 1 indicating the child made an initial movement to the wrong body part but then self-corrected to the correct body part. The HTKS-R has been shown to demonstrate strong reliability and validity in diverse samples of young children (Gonzales et al., 2021; McClelland et al., 2021).

#### HTKS-Kids tablet assessment

The HTKS-Kids tablet-based version of HTKS-R includes two formats where the teacher is first more involved, and then less involved. In the first part, Opposites, the child sat next to the teacher who was holding the tablet, and the child listened to the tablet app instructions (“If I say head, you say toes”), and then stated their answer verbally. Teachers entered on the tablet whether the child's response was head or toes. After these items, the teacher handed the tablet to the child and listened to instructions that were analogous to HTKS-R items, interacting with an animated panda on the touchscreen instead of their own body (i.e., the tablet would say, “If I say tap panda's head, you tap panda's toes”). The teacher remained next to the child to facilitate engagement. HTKS-Kids included four sections with the same rules as in the HTKS-R: Opposites (spoken) and Parts 1, 2, and 3 (child touches panda's body parts instead of their own). HTKS-Kids items were scored 0 (incorrect), 1 (self-correct), 2 (correct) in the Opposites section that was teacher-mediated; and 0 (incorrect) or 2 (correct) in the other (panda) sections.

We created a short HTKS-Kids training video and handout and discussed it with teachers. They were asked to play the HTKS-Kids tablet assessment once with each study child, entering children's first names and last initial. Teachers or the study RA exported the data to a secure folder that only the research team could access. Two teachers accidentally gave the HTKS-Kids assessment to 12 children more than once (from 2 to 5 times). Teachers played HTKS-Kids with 79 children, including 75 children who also took the HTKS-R.

#### Key differences in HTKS-R and HTKS-Kids

We note several key differences between HTKS-R and HTKS-Kids. First, HTKS-Kids removed the gross motor component that is a defining feature of the HTKS-R. Thus, the HTKS-Kids requires children to apply their EF while self-regulating to sit, speak, and hold and touch the tablet on this revised version of the task. Second, we eliminated from 1 to 3 items in each of the four HTKS-Kids sections to reduce overall assessment time and increase engagement; this means there are fewer total HTKS-Kids items (38) than HTKS-R items (59). Third, self-correct scores were not an option for children in the HTKS-Kids panda sections (Parts 1, 2, and 3), because training children on how to change their answer on the tablet was too complicated. Finally, HTKS-R was given by an assessor previously unknown to the child in a quiet area outside the classroom, whereas HTKS-Kids was given by the child's regular teacher in different settings determined by the teacher, including within the classroom while other activity was happening around them. These differences mean that the tasks pose varying EF and self-self-regulatory demands and make a study comparing HTKS-Kids and HTKS-R scores important.

#### Child behavior rating scale

Teachers rated each study child using a 5-pt Likert-style scale on 10 items from the Child Behavior Rating Scale (CBRS; Bronson, 1994) that represent children's ability to demonstrate self-regulation in the complex context of the classroom. Example items from the classroom self-regulation subscale include “observes rules and follows directions without reminders,” and “returns to unfinished task after interruption.” The CBRS classroom self-regulation composite has been shown to be reliable and valid in diverse groups of children (Matthews et al., 2009; Wanless et al., 2011a). Previously reported correlations between CBRS classroom self-regulation and earlier versions of HTKS-R vary; in a preschool sample of 247 children with similar characteristics, the correlation was *r* = 0.35 (Schmitt et al., 2014). The reliability for CBRS items in this study was high at α = 0.95. We obtained CBRS ratings for 80 children and calculated a mean score composite from the 10 items for use in analyses.

### Analytic approach

We used EpiData for paper data entry including double entry of HTKS-R forms and CBRS rating scales, Excel for data management and preparation; and SPSS 27 (IBM Corp, 2021) and Mplus 8.0 (Muthén and Muthén, 1998) with MCAR estimator.

For RQ1: We analyzed only those items that were the same in HTKS-R and HTKS-Kids, with each tasks' score maximum therefore being 76 points for 38 total items. Practice items and test items counted toward this total of 38 items, and all items were included when creating composites. We analyzed both raw HTKS-R scores, and rescaled HTKS-R where for Parts 1, 2, and 3, self-correct scores of 1 were recoded as 2 to match the scale on the HTKS-Kids panda sections. Prior analyses have shown that a self-correct score on HTKS is statistically similar to a score of 2 (Bowles et al., n.d.).^1^ For all analyses comparing HTKS-R and HTKS-Kids task items or composites, we used the rescaled HTKS-R scores. Finally, because separate task sections include different numbers of items, we calculated sum scores but also mean scores to facilitate task and composite comparisons.

For RQ2: To understand sources of variability in HTKS-Kids scores, we performed stepwise regressions where HTKS-Kids sum score was regressed on age, then we added parent education, then we added first language other than English, then we added whether the child was female, and finally we added whether the child was Black or Latino.

For RQ3: To understand whether HTKS-Kids provided different information from teacher-rated classroom self-regulation, we first examined correlations between and mean differences in each measure. We then ran simple *t*-tests for four different groups (female vs. male, Black or not Black, Latino vs. non-Latino, and white vs. non-White) for the normally-distributed CBRS scores. We used the Mann-Whitney *U* statistic for the non-normal HTKS-Kids sum score. Finally, to assess whether any simple mean differences remained statistically significant after adjusting for key background characteristics, we conducted linear regressions in Mplus using the MLR estimator controlling for important background variables and utilizing all available data.

## Results

### RQ1: HTKS-Kids preliminary reliability and validity

We found that item scores were highly similar between the rescaled HTKS-R items and HTKS-Kids assessment. We ran selected pairwise comparison *t*-tests to see whether differences of magnitude 0.20 or above were statistically significant. Children scored the same on analogous task items, except scores were significantly lower on the first three practice items on Part 1 on HTKS-Kids, compared to the same items on the HTKS-R.

#### Range and distribution

Overall, composite and total scores were highly similar between the HTKS-R and HTKS-Kids versions (see Table 1). A small number of children scored at floor (<6% of the sample) on each task version. Of note, HTKS-Kids achieved an important objective for score distribution; only 6% or 5 children scored at floor. Recent work on the HTKS-R with a low-income sample of children in Oregon indicated that 3% of 4-year-old children scored at floor (Gonzales et al., 2021). In this study, both tasks showed a positive skew, meaning that fewer children achieved higher scores and the bulk of the sample scored below the mean. The HTKS-Kids distribution was bimodal, with no scores falling between 25 and 35. While bimodal scores are typical of this assessment (Cameron Ponitz et al., 2008), this pattern of distribution was pronounced with the HTKS-Kids and led to our using non-parametric analyses for subsequent analyses. We employed Mann-Whitney U tests and the MLR estimator in Mplus which is appropriate for non-normally distributed data.

**Table 1:** Composite descriptives by task and task section.

	**Sum**		**Mean**		**SD**		**Max**	
	**Kids**	**HTKR**	**Kids**	**HTKR**	**Kids**	**HTKR**	**Kids**	**HTKR**
Total	21.7	21.8	0.54	0.54	0.47	0.49	1.70	1.90
Opposites	9.9	10.5	1.41	1.51	0.71	0.65	2.00	2.00
Part 1	6.3	6.6	0.52	0.55	0.58	0.65	1.83	2.00
Part 2	3.9	3.1	0.36	0.28	0.61	0.59	2.00	2.00
Part 3	1.6	1.6	0.16	0.16	0.35	0.43	1.40	2.00
	**Skewness**		**Kurtosis**		**% (#) Floor**		**% (#) Ceiling**	
	**Kids**	**HTKR**	**Kids**	**HTKR**	**Kids**	**HTKR**	**Kids**	**HTKR**
Total	0.94	1.35	−0.35	0.88	6 (5)	8 (6)	0	0
Opposites	−0.83	−1.27	−0.78	0.36	9 (7)	9 (7)	47 (37)	42 (33)
Part 1	0.93	1.24	−0.68	0.10	25 (20)	27 (21)	0	3 (2)
Part 2	1.63	1.96	1.44	2.40	67 (53)	77 (60)	6 (5)	1 (1)
Part 3	2.44	2.94	5.26	8.00	76 (60)	83 (65)	0	3 (2)

HTKS-Kids N = 79. HTKS-R (HTKR) N = 78. All values in this table are based on mean scores (with range 0–2) except the first set of descriptives which is for sum scores.

#### Inter-item and test-retest reliability for HTKS-Kids

Our study was not designed to assess reliability of HTKS-Kids, but we calculated alpha values for HTKS-Kids items, and also examined test-retest reliability for the handful of children whose teachers mistakenly gave them HTKS-Kids more than once.

Inter-item reliability for the 38 HTKS-Kids items was excellent, at α = 0.95. Twelve children played HTKS-Kids 2, 3, 4, or 5 times over a 2-week period. Within children, test-retest reliability for the first and second occasions was excellent with Cronbach's alpha of 0.89, and inter-item correlation of 0.80. For these 12 children, the average duration between the 1st and 2nd occasion was 3 days, with a range from 1 to 6 days. Additionally, and for descriptive purposes only given that small numbers of children took HTKS-Kids 3, 4, or 5 times, Table 2 shows every sum score for each child across up to five occasions (T1–T5). With the exception of Child H, whose score improved dramatically after T1, most children scored within a fairly narrow range after repeated attempts.

**Table 2:** Sum scores on HTKS-Kids over up to five occasions within *n* = 12 children.

**Child**	**T1**	**T2**	**T3**	**T4**	**T5**
A	0	4	6		
B	0	1	2		
C	8	6			
D	9	20	16		
E	12	18	20		
F	14	16			
G	14	18	40	34	
H	17	50	60	48	
I	38	40	34		
J	42	58	56		
K	44	36	50	44	42
L	58	44	44		

#### Correlations between HTKS-R and HTKS-Kids

HTKS-R and HTKS-Kids scores were positively correlated at *r* = 0.60; *r* = 0.59 controlling for age. This magnitude is higher than correlations among different self-regulation and EF measures, which tend to fall around *r* = 0.3 or 0.4; one exception is that among 4-year-olds, the HTKS-R correlates above *r* = 0.54 with the Dimensional Change Card Sort (DCCS) task (McClelland et al., 2014). One small study found the correlation between the HTKS given to 25 children by researchers and teachers is *r* = 0.99 (McClelland et al., 2015a; see Table 3).

**Table 3:** Zero-order correlations by task and task section (above diagonal).

	**1**	**2**	**3**	**4**	**5**	**6**	**7**	**8**	**9**	**10**	**11**
1. Kids total	–	0.60	0.63	0.41	0.94	0.60	0.91	0.54	0.80	0.36	0.42
2. HTKS-R total	0.59	–	0.41	0.59	0.63	0.93	0.51	0.91	0.41	0.80	0.41
3. Kids opposites	0.65	0.39	–	0.54	0.46	0.38	0.37	0.25	0.30	0.18	0.34
4. HTKS-R opposites	0.41	0.58	0.51	–	0.33	0.45	0.30	0.31	0.21	0.24	0.33
5. Kids part 1	0.94	0.63	0.50	0.35	–	0.63	0.84	0.60	0.71	0.44	0.38
6. HTKS-R part 1	0.58	0.92	0.36	0.43	0.62	–	0.51	0.80	0.44	0.64	0.39
7. Kids part 2	0.92	0.50	0.39	0.31	0.85	0.50	–	0.50	0.72	0.29	0.35
8. HTKS-R part 2	0.52	0.91	0.23	0.30	0.59	0.80	0.48	–	0.37	0.78	0.36
9. Kids part 3	0.80	0.39	0.32	0.21	0.73	0.41	0.71	0.35	–	0.25	0.31
10. HTKS-R part 3	0.34	0.80	0.17	0.23	0.42	0.63	0.28	0.78	0.24	–	0.31
11. CBRS self-reg mean	0.40	0.38	0.34	0.30	0.38	0.35	0.34	0.34	0.25	0.21	–

Correlations with age partialed out (below diagonal).

Pearson correlations used pairwise deletion. Ns range from 74 to 79 depending on the measures.

All correlations above 0.30 are *p* < 0.01. Correlations 0.24–0.29 are *p* < 0.05. Correlations 0.20 are *p* < 0.10. n = 74.

### RQ2: sources of variability in HTKS-Kids scores

In the first regression model, older children scored significantly higher on HTKS-Kids scores, but the overall model did not explain variance that differed from zero (see Table 4). In each step that included parent education, children whose parents reported higher education levels scored significantly higher on HTKS-Kids, *p* < 0.01. The magnitude of this association was modest: when parents reported 1 SD, or 2.3 years higher than the mean 12.5 years of education, children scored from 0.25 to 0.37 points higher on HTKS-Kids.

**Table 4:** HTKS-Kids sum scores regressed on key child characteristics.

	**Step 1 (*****n*** = **86)**		**Step 2 (*****n*** = **86)**		**Step 3 (*****n*** = **83)**		**Step 4 (*****n*** = **82)**		**Step 5 (*****n*** = **77)**	
	**Coeff**.	***t***	**Coeff**.	***t***	**Coeff**.	***t***	**Coeff**.	***t***	**Coeff**.	***t***
Int.	−2.10	−1.38	−2.95	−2.17^*^	−2.90	−1.99^*^	−2.88	−1.96^*^	−1.80	−1.19
Age	0.22	2.12^*^	0.17	1.64	0.13	1.23	0.13	1.22	0.11	1.03
Par. Edu.			0.29	2.63^**^	0.37	3.95^**^	0.37	3.94^**^	0.25	2.71^**^
Lan. Oth.					0.11	0.88	0.11	0.87	0.21	1.60
Female							0.00	−0.02	0.02	0.18
Black									−0.07	−0.51
Latino									−0.32	−2.53^**^
R-sq.	0.05	1.06	0.13	1.97^*^	0.18	2.77^**^	0.18	2.78^**^	0.17	2.94^**^

Par. Edu., parent education; Lan. Oth., family reported a language other than English as child's first language.

^*^p < 0.05.

^**^p < 0.01.

In the last model we tested, children whose families identified them as Latino scored significantly lower, *p* < 0.05; however, this model did not explain more variance from the model without Black or Latino indicator variables. Thus, we conclude that in this sample, the only meaningful predictor of children's HTKS-Kids score was their parent's level of education.

### RQ3: teacher rated compared to tablet-assessed self-regulation (HTKS-Kids)

HTKS-Kids scores were moderately and positively correlated with teacher ratings of classroom self-regulation, with *r* = 0.40. This is similar to correlations between CBRS and HTKS in other studies, with *r* = 0.29 (Cameron Ponitz et al., 2009) and *r* = 0.35 (Schmitt et al., 2014; see Table 3).

In simple comparisons without predictors, we found no differences by gender in either HTKS-Kids or teacher ratings. However, teachers scored Black children as having about 0.3-point or half an SD lower classroom self-regulation compared with non-Black children, *t* = −2.00, *p* < 0.05; and teachers rated white children about 0.4-point (also half-SD) higher on classroom self-regulation than non-white children, *t* = 2.22, *p* < 0.05. See raw score differences by key groups in Table 5. We also found that Latino children scored 0.4-point lower on HTKS-Kids than non-Latino children, *z* = −2.82, *p* < 0.01.

**Table 5:** Raw score means and SDs for distinct subgroups.

	**HTKS-Kids**					**CBRS**	
	**N**	**M**	**SD**		**N**	**M**	**SD**
Female	41	22.05	19.77	Female	42	3.58	0.78
Male	37	21.32	17.78	Male	42	3.44	0.69
Black	30	20.83	15.75	Black	29	3.24	0.68
White	16	26.50	22.28	White	17	3.84	0.76
Latino	20	13.40	15.91	Latino	21	3.30	0.69

Tables 6A–D show standardized results for the Mplus regressions, which means that the coefficients indicate the percent of SD change in outcome score given a 1 SD increase in the predictor variable. Standardized coefficients can also be compared within the same model. Overall, we found the same pattern of results as with simple group comparisons.

**Table 6A:** No gender differences on HTKS-Kids or teacher-reported CBRS.

	**HTKS-Kids**		**CBRS**	
	**Coeff**.	***t***	**Coeff**.	***t***
Int.	−2.88	−1.96^+^	−1.94	−1.40
Age	0.13	1.22	0.34	3.78^**^
Par. Edu.	0.37	3.94^**^	0.26	3.29^**^
Lan. Oth.	0.11	0.87	0.05	0.52
Female	0.00	−0.02	0.12	1.15
Total r-sq	0.18	2.78^**^	0.23	2.82^**^

N = 78.

Par. Edu., parent education; Lan. Oth., family reported a language other than English as child's first language.

^**^p < 0.01.

^+^*p* = 0.05.

**Table 6B:** Teachers rate Black children lower on CBRS but HTKS-Kids scores do not differ.

	**HTKS-Kids**		**CBRS**	
	**Coeff**.	***t***	**Coeff**.	***t***
Int.	−2.91	−1.99^*^	−1.52	−1.08
Age	0.13	1.21	0.33	3.62^**^
Par. Edu.	0.37	4.00^**^	0.26	3.57
Lan. Oth.	0.12	0.93	−0.03	−0.29
Female	0.12	0.00	0.11	1.08
Black	0.04	0.37	−0.25	−2.41^*^
Total r-sq	0.18	2.85^**^	0.28	3.84^**^

N = 78.

Par. Edu., parent education; Lan. Oth., family reported a language other than English as child's first language.

^*^p < 0.05.

^**^p < 0.01.

**Table 6C:** Latino children score lower on HTKS-Kids but teacher ratings do not differ.

	**HTKS-Kids**		**CBRS**	
	**Coeff**.	***t***	**Coeff**.	***t***
Int.	−1.87	−1.23	−1.10	−0.75
Age	0.11	0.99	0.31	3.40^**^
Par. Edu.	0.26	2.82^**^	0.22	2.30^*^
Lan. Oth.	0.22	1.80^+^	0.12	1.15
Female	0.02	0.13	0.10	0.86
Latino	−0.28	−2.90^**^	−0.16	−1.26
Total r-sq	0.17	2.99	0.20	2.23^*^

N = 78.

Par. Edu., parent education; Lan. Oth., family reported a language other than English as child's first language.

^*^p < 0.05.

^**^p < 0.01.

^+^*p* = 0.07.

**Table 6D:** Teachers rate white children higher on CBRS but HTKS-Kids scores do not differ.

	**HTKS-Kids**		**CBRS**	
	**Coeff**.	***t***	**Coeff**.	***t***
Int.	−2.50	−1.63	−1.60	−1.12
Age	0.12	1.07	0.32	3.57^**^
Par. Edu.	0.30	3.29^**^	0.23	2.77^**^
Lan. Oth.	0.13	1.01	0.09	0.93
Female	−0.01	−0.10	0.13	1.26
White	0.13	1.11	0.29	3.14^**^
Total r-sq	0.15	2.72^**^	0.27	3.13^**^

N = 78.

Par. Edu., parent education; Lan. Oth., family reported a language other than English as child's first language.

^**^p < 0.01.

#### Gender

There were no significant differences by gender in HTKS-Kids or CBRS teacher ratings in the Mplus regressions controlling for age, parent education, and language status (see Table 6A).

#### Black vs. non-Black

There were no differences in HTKS-Kids scores for children their families identified as Black vs. children not identified as Black. In contrast, teachers rated Black children lower on CBRS classroom self-regulation: the coefficient of −0.23 if the child was Black was similar in magnitude to the 0.28 coefficient for parent education (see Table 6B).

#### Latino vs. non-Latino

In regressions controlling for background variables including first language, there were no score differences in teacher-rated CBRS classroom self-regulation, but Latino children scored significantly lower on HTKS-Kids. The magnitude of this difference if the child was identified as Latino, −0.25, was again similar to the coefficient of 0.27 for parent education (see Table 6C).

#### White vs. non-white

There were no differences for white vs. non-white children on HTKS-Kids scores; but teachers rated white children higher on CBRS classroom self-regulation. The coefficient of 0.31 if the child was white was similar in magnitude to the coefficient of 0.24 for parent education (see Table 6D).

## Discussion

The present study examined the initial psychometric properties of a tablet-based version of a popular research measure of self-regulation requiring EF, called HTKS-Kids, including variability, reliability, and validity using comparison with the established HTKS-R measure. We investigated potential sources of difference in children's scores and also examined whether scores on HTKS-Kids correlated with, and provided different information from teacher ratings of these skills in the classroom. We report three main findings. First, the new HTKS-Kids tablet measure of self-regulation facilitated by preschool teachers showed early evidence of reliability and validity. Second, the best predictor of tablet-based HTKS-Kids self-regulation score was parent education. Third, correlation of HTKS-Kids with teacher ratings was moderate; further, teachers rated Black children lower, but white children higher, on classroom ratings, whereas these differences did not appear in children's HTKS-Kids scores.

### HTKS-Kids showed strong internal consistency and correlated with HTKS-R

HTKS-Kids captured individual differences in self-regulation among 4-year-old children. Additionally, a subgroup of children who took the HTKS-Kids more than once within a short time period achieved highly similar scores. Based on correlations with the existing, established measure of self-regulation, HTKS-Kids measured self-regulation: Children who scored higher on HTKS-Kids also scored higher on the original HTKS-R research task, and were rated with better classroom self-regulation by their teachers. These findings all suggest that HTKS-Kids is a promising new tablet-based, direct measure of self-regulation that can be used inside preschool classrooms and facilitated by early childhood teachers. As assessment demands on teachers increase, practical measures that provide direct information on children's skills across a range of school readiness domains become more important (Maves, 2022).

We found very few differences when comparing analogous individual items on the two measures, except children scored lower on the first few HTKS-Kids items where they interacted directly with the tablet, as compared with corresponding items on the HTKS-R. Their lower HTKS-Kids item scores make sense because those particular HTKS-Kids items represent a transition from listening to the teacher and speaking responses, to holding and pressing the tablet screen. This transition may pose higher self-regulatory demands than corresponding items on the HTKS-R where there is a transition from listening/speaking, to listening/responding with gross motor movements without a tablet involved.

The correlation of *r* = 0.60 between the total scores on HTKS-Kids and HTKS-R was not as high as we expected in a task that includes the same items delivered in a different format and setting. As we previously noted, HTKS-Kids included fewer overall items as well as greater variation in how teachers administered the task (inside or outside the classroom, though we do not have this information at the child level). In contrast, the HTKS-R was administered by a researcher unknown to the child, in a relatively quiet space outside the classroom. These differences likely accumulated, resulting in a lower-than-expected correlation. The correlation alone, however, does not mean that HTKS-Kids is not providing valuable information about children's self-regulation; as noted previously, the HTKS-R and DCCS are correlated around 0.60 and both are considered robust measures of self-regulation that require children to apply EF processes to their behavior (McClelland et al., 2014). Future research should continue to test HTKS-Kids and HTKS-R associations with larger and more diverse samples of children.

### Parent education explained the most variance in children's HTKS-Kids scores

The best predictor of children's HTKS-Kids scores was parent education. Parent education is a proxy for early learning experiences and resources, which are consistently linked to EF development and overall self-regulation (Davis-Kean, 2005; Waters et al., 2021). Child age was not a significant predictor of HTKS-Kids scores, but this may be due to the combination of the relatively narrow variation in age and the sample size under 80. Importantly, we did not find that whether children were identified by their parents as Black or white explained any variance in HTKS-Kids scores. Given that race is a social, not biological construct, it is encouraging that children performed similarly on the new HTKS-Kids assessment regardless of this identity variable.

Although sub-sample sizes were small, we found that non-Black, Latino-identified children had lower HTKS-Kids scores, even after controlling for parent-reported child first language status and parent education. Another study found that among low-income children, Latino children showed less developed self-regulation and improved more slowly over time as compared to white children (Wanless et al., 2011b). We note that our analyses are based on small subgroup numbers: only eight of 17 Latino children were identified by their families as speaking Spanish as their first language, with the other nine Latino children identified with a first language of English. All our study children were given only the English version of HTKS-Kids, so it is not possible to address whether Spanish-language administration may have improved scores. Therefore, the extent to which language and culture played a role in HTKS-Kids performance was not possible to fully explore in this study but needs to be better understood.

### Is HTKS-Kids providing different information for Black children than teacher ratings?

As in other studies including the HTKS (Cameron Ponitz et al., 2009; Schmitt et al., 2014), HTKS-Kids was also moderately positively correlated with teacher ratings of self-regulation. Modest or moderate positive correlations are common among measures of regulatory processes that vary in design features (Rimm-Kaufman et al., 2009; Vitiello et al., 2011; McClelland et al., 2014), such as different formats (e.g., paper or tablet), settings (e.g., individualized or naturalistic), response modalities (e.g., gross motor actions, points, or key presses), and administrators (researchers or teachers). Teacher ratings are based on their observations and interactions with children in their classroom over several weeks or months, and the CBRS items in particular asked teachers for their aggregate impressions on how well children manage attention, materials, and behavior across various learning situations. On the other hand, HTKS-Kids performance reflects a score derived from a single individualized assessment where the teacher was present in one-on-one interaction. Distractions from peers did vary, because some teachers reported that they took children outside the classroom to play HTKS-Kids, though we did not collect information on each child's specific assessment context. Teacher discretion on administration setting is an important part of naturalistic assessment, but this variability along with the other differences between CBRS ratings and HTKS-Kids are likely sources of other findings based on child demographic characteristics. Specifically, teachers rated children similarly regardless of Latino ethnicity, but we found other differences in teacher ratings of children's classroom self-regulation if the child was Black. That is, teachers rated white children more favorably than non-white children, and Black children worse than non-Black children.

In explaining these findings, the literature on implicit bias must be considered along with the aforementioned discussion of assessment differences. First is the possibility that there is some “true” difference in children's self-regulatory behaviors in the classroom; for example, perhaps Black children were able to be as successful as non-Black children in the more structured HTKS-Kids context, but exhibited greater levels of distraction in typical classroom learning settings, leading to lower teacher ratings. We note that “true” self-regulation differences could arise from a classroom system that is less supportive for Black children than for white children. That is, teachers may interact with Black and white children differently in regular classroom interactions, which could lead to Black children responding and self-regulating differently. For example, teachers might unconsciously use a less warm tone with Black children; this inconsistent emotional support could activate the child's nervous system and lead to problems self-regulating as children struggle to focus on the task at hand, possibly worrying about their teachers' attitude toward them (Curby et al., 2013). Similarly, Black children may exhibit some behaviors—such as physical and vocal expressiveness—that Boykin explains are a rich legacy of their African American heritage (Boykin and Allen, 1988), but which may be incorrectly interpreted as indicators of poor self-regulation by some teachers. As a reminder, over 80% of this study's teachers identified as white. Finally, it is possible that teachers see the same behaviors differently depending on children's ethnicity. For example, literature supports the idea that teachers discipline Black children more harshly for offenses compared with white children (Ispa-Landa, 2018).

One of the key goals of this study and the broader program of research is to use technology to develop more equitable assessment for young children. The possibility of HTKS-Kids providing information about children that could enrich their teachers' preexisting views of their potential, based on conclusions from observing the child in traditional classroom situations alone, is an important one in this broader context.

### Limitations and future directions

Overall study results were preliminary but promising, and the small sample size necessitates further work to establish psychometric properties and relatedly (and perhaps most importantly), to identify age-based norms and/or screening cutoffs so that HTKS-Kids can be more useful to early childhood programs. While HTKS-Kids is based on the well-established research instruments HTKS and HTKS-R (Cameron Ponitz et al., 2009; McClelland et al., 2014; Gonzales et al., 2021), HTKS-Kids is also different in meaningful ways, given that it involves children interacting with a tablet, and is teacher-facilitated for use in typical classroom settings. Thus, similar research using HTKS-Kids with a larger sample and to continue to examine validity using more than just the HTKS-R, including other measures of self-regulation and early academic skills, is needed. Statistically, a larger sample can enable analysis of items to see how well HTKS-Kids captures child and item differences, norming to identify average scores for children of a given age, and/or cutoff scores that indicate further assessment for possible intervention is needed. More data with both younger and older children are needed. Because the EF processes that underlie a child's ability to self-regulate are implicated among students with ADHD (Barkley, 2004), a modified, age-appropriate version for older students could provide school psychologists for an additional tool for their work with this population.

Given the cultural-embeddedness of self-regulation and its assessment, broadening the socio-demographic characteristics of children given HTKS-Kids is perhaps the most important. It is promising that the HTKS has been translated into 28 languages and used worldwide, and a meta-analysis indicated no differences in how well HTKS predicted young children's academic achievement by country or cultural context (Kenny et al., 2023). Collecting data with a larger sample of racially diverse children can help establish whether HTKS-Kids could mitigate implicit bias against Black children, which may contribute to the differences we found in teacher ratings of children's classroom self-regulation. Future research should also examine the HTKS-Kids in larger samples of multi-lingual children, perhaps using a screening tool to determine a child's need for Spanish language assessment, and subsequently, a Spanish version of HTKS-Kids. Other potential directions include offering children a choice about their language of assessment, and/or incorporating symbols, which may help broaden the task beyond English- and Spanish-speakers.

Future applications for HTKS-Kids are broad and could have major impact. Within early childhood systems, screenings help provide children access to early intervention services (AAP Council on Early Childhood and AAP Council on School Health, 2016; Bertram and Pascal, 2016). One future longitudinal study could examine the potential for HTKS-Kids to predict referral to special education or intervention services. HTKS-Kids was also designed to be incorporated into a holistic assessment system, specifically Cognitive Toybox (cognitivetoybox.com), which provides observation and individualized game-based assessment measures across whole child development. This design allows HTKS-Kids to be used in conjunction with other academic and non-academic measures to achieve a full understanding of a child's school readiness (Tripathy et al., 2020).

A strong and scalable instrument that measures self-regulation with reliability and validity and that is both child- and teacher-friendly could change the face of kindergarten entry assessment. Common observational tools like Teaching Strategies Gold (TS Gold) are resource-intensive, and misuse of TS Gold and other early childhood assessment tools is common (Ackerman and Lambert, 2020; Olson and Lepage, 2022; Cameron et al., 2023). And as screeners grow more widespread—used in more than half of U. S. states in 2022—currently available tools like the Brigance remain focused on academic and related skills, which are highly dependent on family resources and are not always culture-fair (Olson and Lepage, 2022). Yet EF processes and the overall self-regulation it supports form the foundation of whether children can learn from academic opportunities (Blair and Ku, 2022). Self-regulation is also part of social-emotional learning (SEL) which is increasingly recognized as critical to support. Tellingly, in our collaborations, programs have had to supplement Creative Curricula, which is used by the large majority of Head Start programs, with other programs such as Second Step that more intentionally support SEL. TS Gold also poses heavy burdens on teachers which can take away from their time to interact effectively with children (Kim, 2016; Cameron et al., 2023). In other words, both kindergarten and preschool programs stand to benefit from a child-friendly, teacher-friendly, scalable tablet-based assessment of self-regulation requiring EF.

## Conclusion

Equitable direct assessment of self-regulation is increasingly sought by early childhood systems. Tablet-based assessments can directly measure children's skills in several learning domains and reduce teacher burden. This study suggests that the HTKS-Kids tablet-based self-regulation assessment requiring EF captures individual differences among children with item scores, variability, and floor effects similar to the original HTKS-R task; measures skills that are similar to this established research task; positively relates with teacher ratings of children's classroom skills; and provides a different picture from teacher ratings of children's classroom self-regulation, which was especially evident for Black children. Assessing the potential of children from historically oppressed groups is crucial equity work, and practical tools can support often-under resourced early childhood professionals and programs. Future efforts should further test and refine HTKS-Kids. These early results point to the potential for equitable direct assessment of self-regulation, a capacity that forms the foundation for children's success in and beyond school.

## Data availability statement

The original contributions presented in the study are included in the article/supplementary material, further inquiries can be directed to the corresponding author.

## Ethics statement

The study involving humans was approved by University at Buffalo Institutional Review Board. The study was conducted in accordance with the local legislation and institutional requirements. Written informed consent for participation in this study was provided by the participants' legal guardians/next of kin.

## Author contributions

CC created the HTKS-Kids items for use on a tablet, conceptualized the manuscript, oversaw data collection, performed all analyses, drafted Results and Discussion sections, and readied the paper for publication. MM helped to create the HTKS-Kids items for use on the tablet, helped design the study and craft research questions, advised on analyses, and refined all manuscript sections. TK contributed to Cognitive Toybox programmed HTKS-Kids for use on the tablet and helped to refine all manuscript sections. KS performed most of the data collection and data entry and drafted the Method section. TL drafted the literature review and helped refine the Discussion section. All authors contributed to the article and approved the submitted version.

## Footnotes

^1^Bowles, R. P., McClelland, M. M., Cameron, C. E., Acock, A. C., Montroy, J. J., and Duncan, R. J. (n. d.). *A Rasch Analysis of the Head-Toes-Knees-Shoulders (HTKS) Measure of Behavioral Self-Regulation*. (Unpublished).
